# The role of *BoFLC2* in cauliflower (*Brassica oleracea* var. *botrytis* L.) reproductive development

**DOI:** 10.1093/jxb/eru408

**Published:** 2014-10-28

**Authors:** Stephen Ridge, Philip H. Brown, Valérie Hecht, Ronald G. Driessen, James L. Weller

**Affiliations:** ^1^School of Plant Science, University of Tasmania, Private Bag 55, Hobart, Tasmania 7001, Australia; ^2^School of Medical and Applied Sciences, CQUniversity, Bundaberg Campus, Locked Bag 3333, Queensland 4670, Australia; and Queensland Government Department of Agriculture, Fisheries and Forestry, Bundaberg Research Station, Ashfield Road, Kalkie, Queensland 4670, Australia; ^3^Rijk Zwaan Breeding B.V. De Lier, 2678 ZG, The Netherlands

**Keywords:** *BoFLC*, *BoFT*, *BoVIN3*, *Brassica oleracea*, cauliflower, expression, flowering time, vernalization.

## Abstract

In agricultural species that are sexually propagated or whose marketable organ is a reproductive structure, management of the flowering process is critical. Inflorescence development in cauliflower is particularly complex, presenting unique challenges for those seeking to predict and manage flowering time. In this study, an integrated physiological and molecular approach was used to clarify the environmental control of cauliflower reproductive development at the molecular level. A functional allele of *BoFLC2* was identified for the first time in an annual brassica, along with an allele disrupted by a frameshift mutation (*boflc2*). In a segregating F_2_ population derived from a cross between late-flowering (*BoFLC2*) and early-flowering (*boflc2*) lines, this gene behaved in a dosage-dependent manner and accounted for up to 65% of flowering time variation. Transcription of *BoFLC* genes was reduced by vernalization, with the floral integrator *BoFT* responding inversely. Overall expression of *BoFT* was significantly higher in early-flowering *boflc2* lines, supporting the idea that *BoFLC2* plays a key role in maintaining the vegetative state. A homologue of *Arabidopsis VIN3* was isolated for the first time in a brassica crop species and was up-regulated by two days of vernalization, in contrast to findings in *Arabidopsis* where prolonged exposure to cold was required to elicit up-regulation. The correlations observed between gene expression and flowering time in controlled-environment experiments were validated with gene expression analyses of cauliflowers grown outdoors under ‘natural’ vernalizing conditions, indicating potential for transcript levels of flowering genes to form the basis of predictive assays for curd initiation and flowering time.

## Abstract (precis)

Genetic, association, and expression studies indicate that a mutation in *BoFLC2* explains much of the variability in cauliflower curd formation, curd growth rate, and flowering time.

## Introduction

The transition from vegetative to reproductive growth is a critical phase in the life cycle of flowering plants, especially in monocarpic species, whose life cycle only allows a single opportunity to sexually reproduce. Timing of the transition to flowering depends on integration of endogenous factors such as leaf number and gibberellin (GA) biosynthesis (Bernier and Perilleux, 2005; Mutasa-Göttgens and Hedden, 2009) and environmental cues such as photoperiod, light quality, temperature, and stress (Cerdán and Chory, 2003; Yaish *et al.*, 2011). The biological function of endogenous requirements is to prevent the plant from flowering before it has accumulated the necessary reserves required for energy-intensive reproductive development (Matsoukas *et al.*, 2012). Environmental cues serve to synchronize the flowering of conspecific, cross-pollinating plants, and also ensure that flowering occurs when conditions are conducive to fruit set and seed production (Salomé *et al.*, 2011).

Although these flowering time regulatory mechanisms have developed to ensure reproductive success in nature, it is often necessary to overcome or otherwise manage them in agricultural systems. For example, cultural practices are often focussed on preventing or delaying flowering in crops grown for vegetative organs. Management of flowering time is also essential where crops are produced for reproductive structures to ensure development and maturity of these organs occurs during favourable environmental conditions. Ensuring synchronous flowering between parent lines with markedly different flowering phenology in the production of hybrid vegetable seed is a third classic example of the need to understand and modify the timing of natural flowering behaviour in agricultural crops.

Molecular technology presents an opportunity to understand and manage reproductive development in such production environments. Prospects for doing so have benefited greatly from recent work with the model species *Arabidopsis thaliana*. Over the last decade, genetic analysis in *Arabidopsis* has identified a large number of flowering genes, enabling the development of a model describing the genetic and biochemical pathways that regulate floral induction through both endogenous signals and environmental cues such as vernalization, temperature, and photoperiod (Andrés and Coupland, 2012; Kumar *et al.*, 2012). Many of the genes and genetic pathways involved in *Arabidopsis* flowering have been conserved to some degree in other plant species. Consequently, there exists great potential to extend the molecular model proposed for the control of flowering in *Arabidopsis* to species of economic importance such as the closely related *B. oleracea*, which includes crops such as cauliflower (var. *botrytis*). Temperature is the main environmental factor influencing reproductive behaviour in this species, with vernalization requirements ranging from near-obligate (e.g. cool-temperate spring cultivars) to virtually none (e.g. tropical cultivars) (Friend, 1985). In vernalization-responsive cultivars, temperature affects important processes such as induction of the edible “curd” and subsequent elongation of inflorescence stems. A better understanding of the molecular and genetic factors regulating vernalization-induced flowering would potentially enable the development of strategies for predicting and managing flowering in a production context, and would also assist breeders seeking to extend the geographical range of *B. oleracea* cultivation.

An early attempt to extend knowledge of *Arabidopsis* regulatory networks to *Brassica* crop species involved the identification of flowering time QTLs in the *B. napus* and *B. rapa* chromosomal regions collinear to the chromosomal position of *Arabidopsis FLOWERING LOCUS C* (*FLC*) (Osborn *et al.*, 1997). In *Arabidopsis*, this gene plays a key regulatory role in the vernalization pathway, encoding a MADS-box protein that binds directly to a region of DNA in the first intron and promoter of *FLOWERING TIME* (*FT*) and *SUPPRESSOR OF OVEREXPRESSION OF CO* 1 (*SOC1*), respectively (Michaels and Amasino, 1999; Sheldon *et al.*, 1999; Helliwell *et al.*, 2006). This prevents transcription of the *FT* and *SOC1* integrator genes, thereby inhibiting flowering. At vernalizing temperatures, genes including *VERNALIZATION INSENSITIVE 3* (*VIN3*), *VERNALIZATION1* (*VRN1*), and *VRN2* act together to physically repress *FLC* mRNA transcription and translation, thereby allowing expression of *FT* and *SOC1* (Sung and Amasino, 2004a, b; He and Amasino, 2005).

Subsequent comparative studies in *Brassica* crop species have enabled the isolation of multiple copies of *Arabidopsis FLC* homologues in several *Brassica* crop species including five in *B. napus* and four in *B. rapa* and *B. oleracea* (Tadege *et al.*, 2001; Schranz *et al.*, 2002; Okazaki *et al.*, 2007). In *B. napus,* all five copies have been characterized, with both expression studies and complementation studies in transgenic *Arabidopsis* supporting their conserved function. Genetic and transcriptional studies in *B. rapa* have provided evidence that, of the *FLC* genes, *BrFLC2* plays the most important role in the regulation of flowering time (Li *et al.*, 2005; Kim *et al.*, 2007; Zhao *et al.*, 2010; Xiao *et al.*, 2013).

There has been comparatively little characterization of *FLC* genes in *B. oleracea.* A study by Okazaki *et al.* (2007) involving biennial, annual, and rapid-cycling forms of *B. oleracea* found that a frameshift mutation in exon 4 resulting in an in-frame stop codon of *BoFLC2* was associated with early flowering behaviour. An earlier study by Lin *et al.* (2005) showed that cabbage (*B. oleracea* var. *capitata*) *BoFLC2* transcription (referred to in that paper as *BoFLC4-1*) was regulated by vernalization in a conserved manner, and also isolated a cabbage homologue of the important flowering time integrator gene *FT* and found that expression of this transcript in cabbage apices was inversely related to *BoFLC2* expression.

These findings suggest that *BoFLC2* functionality may underpin important differences between annual and biennial brassica cultivars, particularly with respect to flowering time. However, no research has been conducted to demonstrate the contribution of this gene to flowering time within either annual or biennial brassica types, both of which have broad flowering time ranges across cultivars. In this study, we evaluated the contribution of *BoFLC2* allelic variation to curd initiation and flowering time variation in both segregating populations and inbred populations of annual cauliflower (*B. oleracea* var. *botrytis*) parent lines. Differences in vernalization response and flowering time were investigated at the molecular level by monitoring the transcriptional dynamics of key genes in response to a range of artificial and natural vernalization treatments. Finally, seasonal changes in gene expression patterns were examined in cauliflowers grown outdoors under natural vernalizing conditions.

## Materials and methods

### Plant material

Fifty four homozygous inbred cauliflower proprietary parent lines were provided by vegetable seed breeding company Rijk Zwaan. These lines had been grouped into five flowering classes by Rijk Zwaan breeders, based on time from planting to full flower under field conditions in the southern Netherlands: early (50–70 days); medium early (70–80 days); medium late (80–120 days); late (120–180 days); very late (180–250 days). Supplementary Table S1 summarises the breeders’ information available for these lines.

Two segregating F_2_ populations were established by crossing late-flowering cauliflower proprietary parent lines A and C with early-flowering proprietary parent lines B and D, respectively. Under field conditions present at the Rijk Zwaan Research and Development facility (Fijnaart, the Netherlands), both late-flowering lines had a flowering time of approximately 120 days, and both early-flowering lines flowered after approximately 70 days. Line A had been inbred for two generations (S_2_), line B had been inbred for four generations (S_4_), and lines C and D had been inbred for six generations (S_6_). The F_1_ plants arising from these two crosses were manually self-pollinated to generate F_2_ seed from the A×B and C×D crosses.

### Growth conditions and experimental design

Three of the outdoor experiments were field-based and one was pot-based. The field-based flowering time trials were located at Fijnaart (the Netherlands), Oyster Cove (southern Tasmania), and Forthside (northern Tasmania). At each field site, glasshouse-raised seedlings were transplanted into the soil at a spacing of 45–50cm between plants and 75–80cm between rows. At Fijnaart, 30 seedlings from each of the A, B, C, and D parent lines were planted into the field in April 2008 along with approximately 380 seedlings from each of the A×B and C×D F_2_ populations. At Forthside, a selection of 15 cauliflower parent lines with flowering behaviour ranging from early to very late was sown in 2007. Time of sowing and transplanting was varied according to flowering behaviour in an attempt to achieve uniform curd initiation and flowering between lines; later-flowering lines were sown in mid-summer (late January–early February), and earlier-flowering lines were sown at the end of summer (late February). At Oyster Cove, 50 out of the 54 cauliflower parent lines were transplanted (omitting four lines that failed to germinate) in April 2009. In both Forthside and Oyster Cove trials, three replicates of each parent line were used with approximately ten plants per replicate and both sites were set out in a randomized complete block design.

The pot-based outdoor flowering time trial was conducted at the University of Tasmania’s Horticultural Research Centre (Sandy Bay, Tasmania). Fifty seedlings of a late-flowering (*BoFLC2*) parent line and 50 seedlings of an early-flowering parent line (*boflc2*) were raised in the glasshouse before being transplanted into 4.5 l pots. To facilitate normal plant growth and development and to synchronise flowering between the two lines, the *BoFLC2* line was sown and transplanted earlier than the *boflc2*. Seeds of the *BoFLC2* line were sown in late summer before transplanting outside approximately six weeks later in early autumn. Seeds of the *boflc2* line were sown in early autumn, two weeks later than the *BoFLC2* line, before transplanting outside approximately seven weeks later in mid-autumn.

In the pot-based, glasshouse flowering time trial, 50 of the 54 cauliflower parent lines were transplanted (omitting four lines that failed to germinate) in April 2009. Seven replicates of each line were transplanted 4.5 l pots. Plants were grown under natural light and photoperiod conditions.

Three controlled environment vernalization experiments were conducted in growth cabinets at the School of Plant Science, University of Tasmania. In each case, plants were grown in 2-l pots under a mixture of 36W cool white fluorescent lamps and 60W incandescent lamps with an output of 100 µM m^–2^ s^–1^ as part of a 16h photoperiod regime. In the first experiment, seedlings of the romanesco *BoFLC2* cauliflower parent line 1 classed as ‘late’ flowering were grown at 22 °C. At ten weeks of age, a proportion of these plants were transferred to 5 °C growth cabinets. Plants from both groups were sampled for measurement of gene expression every two weeks until 16 weeks of age. In the second experiment, plants from four *BoFLC2* parent lines (2, 12, 24, and 51) and four *boflc2* parent lines (17, 40, 48, and 50) were grown at 22 °C. At ten weeks of age, a proportion of these plants were transferred to 5 °C, 10 °C, and 15 °C growth cabinets. Gene expression was measured at 3, 6, 10, and 15 weeks of age. In addition, plants vernalized at 5 °C were sampled for gene expression 2, 7, and 21 days after vernalization. In the third experiment, seeds of the same romanesco variety as above were grown at 22 °C. Plants aged 1–12 weeks were exposed to one- and three-week vernalization treatments at 5 °C.

### Phenotyping

A semi-quantitative scale of 1–6 was constructed to allow consistent assessment of curd developmental stage across experiments. Stage 1 corresponded to a curd that is visible without damaging excavation amongst apical leaves, and that was compact, with no discernible elongation of peduncles. Stage 2 corresponded to a ‘loose’ curd that was beginning to ‘break’. Stage 3 corresponded to a curd whose early-order inflorescence branches were elongating, but where individual flower buds were not formed, or were rudimentary. Stage 4 curds had fully formed flower buds (similar to the individual buds of a broccoli head). Stage 5 corresponded to a curd that had one or more flowers fully open, but less than twelve flowers fully open. Stage 6 was the highest developmental category, with curds that had more than 12 flowers open, and the possible presence of seed pods. Images of these six stages are provided in Supplementary Fig. S1.

### DNA extraction and genotyping

In the segregating F_2_ population, DNA was isolated from 8mm young leaf discs using a mag Plant DNA Isolation Kit (AGOWA) in conjunction with a KingFisher magnetic particle processor (Thermo Scientific) according to manufacturer’s specifications. For all other experiments, DNA was extracted using a modified CTAB protocol. CAPS and size markers were developed to genotype *BoFLC2* and *BoFLC4-*1. Details of these markers are shown in Supplementary Table S2.

### Gene expression

Harvested tissue consisted of young, non-fully expanded leaves and apices. Plants at a physiological age of one week did not possess any true leaves, so pooled tissue samples from multiple comprising cotyledon/apex tissue were harvested instead, as with Lin *et al.* (2005). Samples were frozen in liquid nitrogen and total RNA extracted using the SV total RNA Isolation System (Promega) according to manufacturer’s instructions. Reverse transcription was performed in 20 µl with 1 µg of total RNA using the MMLV High Performance Reverse Transcriptase System (Epicentre Biotechnologies) according to manufacturer’s instructions. RT negative (no enzyme) controls were included to monitor for gDNA contamination. First-strand cDNA was diluted five times and 2 µl was used in each real-time PCR reaction. Real-time PCR reactions using SYBR green chemistry (Sensimix) were run for 50 cycles in a Rotor-Gene RG3000 (Corbett Research). Two technical replicates and three biological replicates were performed for each sample unless otherwise stated. Transcript levels for experimental genes were normalised against the constitutively-expressed reference gene ACTIN (ACT) using nonequal efficiencies (Pfaffl, 2001). Primer details are included in Supplementary Table S2.

## Results and discussion

### BoFLC2 is strongly associated with flowering behaviour in annual and biennial *Brassica* parent lines

Cauliflower inbred parent lines representing a diverse range of flowering types were screened for the presence of the single base deletion in exon 4 of *BoFLC2* first described by Okazaki et al (2007). Of the 54 parent lines, 33 carried the non-functional allele (*boflc2*) and 21 carried *BoFLC2*. The *BoFLC2* genotype was strongly associated with delayed flowering as categorized by Rijk Zwaan breeders (Supplementary Table S1), with a higher proportion of lines carrying functional alleles found in classes defined by later flowering time (Supplementary Fig. S2), consistent with the role of *BoFLC2* as a flowering inhibitor. These findings constitute the first record of functional *BoFLC2* alleles in cauliflower varieties, and support the idea that the previously described *boflc2* mutation (which is predicted to result in a loss of function owing to a premature termination codon) is a critical determinant of flowering time within the annual brassica subgroup.

Identification of a strong association between *BoFLC2* allelic variation and floral induction in ‘natural’ populations of cauliflower inbred parent lines is potentially of great practical and commercial value. The *boflc2* marker could be used for germplasm screening and reliable selection of appropriate cauliflower genotypes in breeding programmes, thereby reducing the need for lengthy field assessments. The number of crosses necessary in doubled haploid (DH) breeding programmes could be reduced by using this marker to help select parent lines with suitable combinations of alleles. In some cases, *BoFLC2* could be modified or transformed to alter timing of floral induction without significant negative pleiotropic effects on yield or general fitness. For example, Kim *et al.* (2007) reported a substantial delay in Chinese cabbage (*B. rapa* ssp. *pekinensis*) floral induction by overexpression of *FLC*. In *B. oleracea* cultivars where delayed curd induction or flowering is sought, such an approach may find a similar application.

### Glasshouse and field trials validate the association of BoFLC2 with reproductive behaviour

**Fig. 1.**
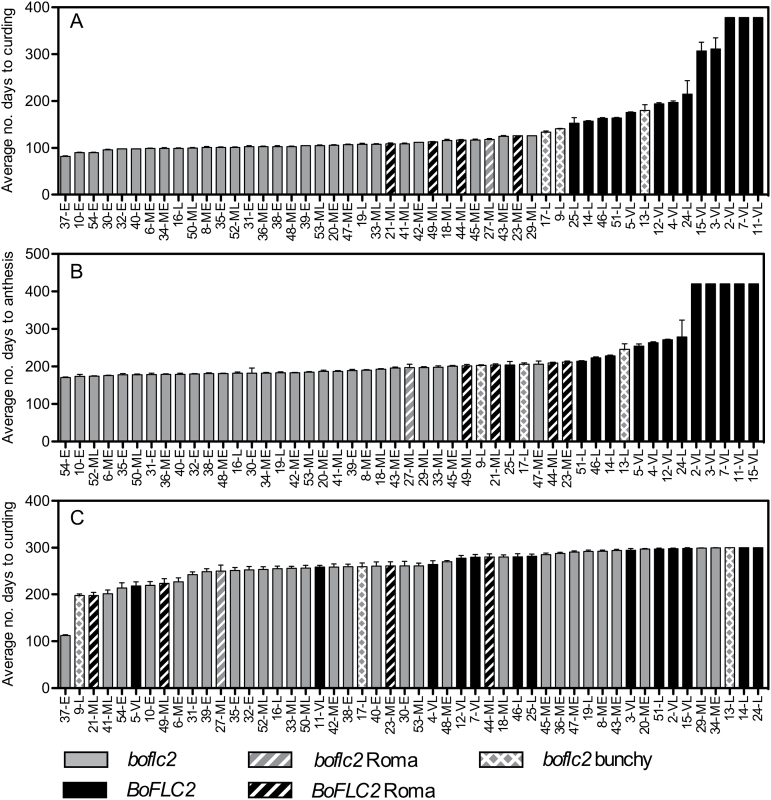
Curding and flowering time of *BoFLC2* and *boflc2* cauliflower parent lines grown under glasshouse and Oyster Cove field conditions. Grey and black columns denote mutant (*boflc2*) and functional (*BoFLC2*) lines, respectively. Columns with stripes represent romanesco parent lines, and columns with spots denote *boflc2* lines that formed a tightly bunched ‘head’ of leaves before curd formation. The flowering class of each parent lines is shown beside each line number, where E=early; ME=medium early; ML=medium late; L=late; and VL=very late. Error bars denote the standard error of the mean. For plants grown in pots under glasshouse conditions, the average number of days from sowing to curding and anthesis is shown in (A) and (B), respectively. Values are the average of seven replicates. Where plants did not initiate visible curds, they were assigned a maximum value of 378 days, and where plants did not flower, they were assigned a maximum value of 420 days. (C) Average number of days from sowing to curding is shown for plants grown under field conditions. Values are the average of up to 30 plants (three replicates with ten duplicates in each replicate), with dead or missing plants excluded from the analysis. Where plants did not initiate visible curds, they were assigned a maximum value of 300 days.

In the pot-based glasshouse flowering time trial, curd initiation data for the 50 different parent lines sown was broadly consistent with the classifications provided by Rijk Zwaan pre-breeders (Fig. 1A). Despite considerable differences in genetic background between these parent lines, a clear advance in curd initiation time was observed in the 33 *boflc2* lines relative to 17 *BoFLC2* lines (108.6±2.1 vs 213.9±9.0 days; *P*<0.0001). In the initial screen of cauliflower parent lines, the presence of functional *BoFLC2* alleles in lines classed as medium early or medium late, and the relatively large number of *boflc2* genotypes in the late class seemed somewhat incongruous (Supplementary Fig. S2). This trial revealed that in most cases, the apparent incongruity in flowering time could be explained by the distinct phenotypes of the lines in question. Four of the earliest curding lines carrying *BoFLC2* were of the ‘romanesco’ variety, with fractally arranged pyramidal curds (Kieffer *et al.*, 1998). Three of the latest *boflc2* lines formed small, tightly bunched ‘heads’ of leaves before detection of a visible curd. This apical morphology made it difficult to routinely distinguish the point at which curds were formed, and complicated the relatively simple *BoFLC2* effect observed in field trials.

As with curd initiation, there was a clear separation of *BoFLC2* and *boflc2* groups based on flowering time with the exception of romanesco and bunchy parent lines (Fig. 1B). *BoFLC2* (including romanesco) lines took an average of 14 weeks longer than *boflc2* (including ‘bunchy’) lines to reach full anthesis (flowering stage 6; 286.1±8.0 vs 188.8±1.1 days; *P*<0.0001). Curd initiation time was usually strongly indicative of flowering time, but some lines that initiated curds at a young age remained in the arrested phase and did not proceed to flowering for a considerable length of time (e.g. line 47). Other parent lines that initiated curds very early were slow to flower (parent line 30) or failed to flower altogether (parent line 37) because of poor growth. Therefore, the distinction in both curd initiation and flowering time between *BoFLC2* and *boflc2* parent lines is sharp, allowing for the deceptive ‘transition’ which is primarily due to parent lines with unusual curd initiation behaviour that complicates the *BoFLC2* gene effect.

In the Oyster Cove field trial, plant growth was compromised by exceptionally wet and cold conditions leading to poor plant growth and high attrition, particularly among the *boflc2* lines that are adapted to warmer climates and lower latitudes. Nevertheless, the average number of days required for curd formation was significantly different (*P*<0.0001) between the surviving *BoFLC2* and *boflc2* genotypes (271.8±1.9 and 254.0±1.8 days, respectively), but the difference between the two genotypes was much less clear than in the glasshouse trial. (Fig. 1C). Allowing for the vagaries associated with the production of cauliflowers in environments to which they were unsuited, these trials not only validated the Rijk Zwaan categories, but also enabled identification of the physiological basis for inconsistencies in flowering behaviour between the two genotypes and demonstrated that variation at the *BoFLC2* locus is a fundamental determinant of flowering time.

### Variation at the *BoFLC2* locus contributes to flowering time and curd size in two segregating F_2_ populations

The correlation of *BoFLC2* with flowering time across a diverse range of parent lines suggests that this gene is an important contributor to reproductive behaviour within annual brassicas. To investigate this contribution more precisely, flowering time and curd development were assessed under field conditions in Fijnaart, the Netherlands in populations segregating for functional and mutant *BoFLC2* alleles derived from crosses between two members of the same group (cauliflower × cauliflower). To do this, two different late (lines A and C) and early (lines B and D) proprietary parent lines with suitable commercial qualities were selected for crossing. The *BoFLC2* genotype of these lines was confirmed, showing that the late and early parents had the expected homozygous *BoFLC2* (+/+) and homozygous *boflc2* (–/–) genotypes, respectively.

Genotyping of the A×B F_2_ population revealed a segregation of 102:175:97 (*BoFLC2*:het:*boflc2*) at the *BoFLC2* locus. This conforms to the expected theoretical 1:2:1 distribution ratio of 93.5:187:93.5 (df=2; χ^2^_crit_ with α at 0.01=9.21; χ^2^_obs_=1.67). In the C×D F_2_ population, a segregation of 120:186:71 (*BoFLC2*:het:*boflc2*) was observed, representing a significant departure from the theoretical expected distribution ratio of 94.25:188.5:94.25 (df=2; χ^2^_crit_ with α at 0.01=9.21; χ^2^_obs_=12.80), possibly owing to gametic selection for the functional allele.

**Fig. 2.**
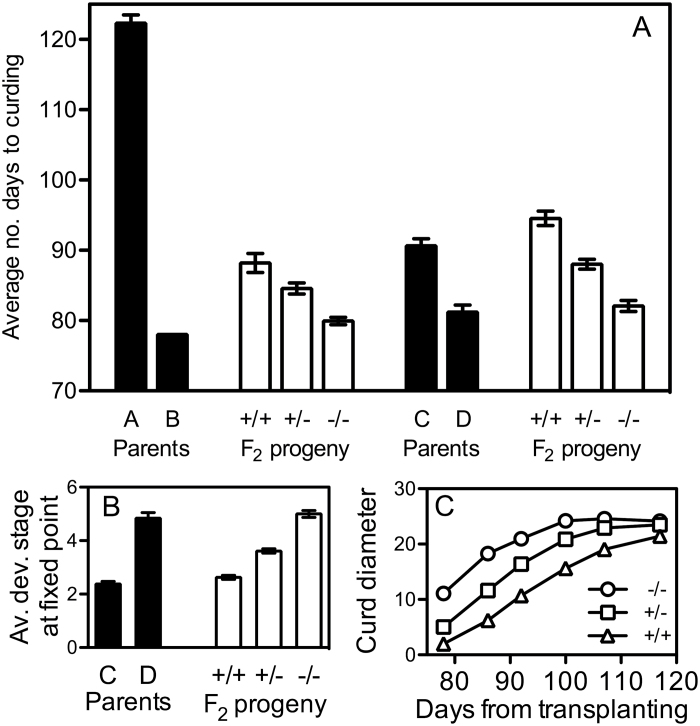
Reproductive behaviour and curd size in segregating F_2_ populations. The average number of days from transplanting until curds were visible in parent lines and F_2_ populations is shown in (A). Plants that had not produced visible curds after 117 days were given a maximum score of 127 days to differentiate them from those plants for which curds were first recorded on the 117th day. In (B), the average developmental stage (c.f. Supplementary Fig. S1) of curds at a fixed point (117 days after transplanting) is shown for C and D parents and F_2_ segregants. Lower developmental stage values represent less-developed curds. In (C) average curd sizes in the C×D segregating F_2_ population are displayed. In all graphs, +/+ denotes plants homozygous for *BoFLC2*, –/– denotes plants homozygous for *boflc2*, and +/– denotes heterozygotes. Error bars represent the standard error of the mean.

Figure 2A shows that in both the A×B and the C×D segregating F_2_ populations, the average length of time from transplanting until curd visibility was significantly different between parent lines and across the three *BoFLC2* genotypes (*P*<0.0001). In the A×B F_2_ population, the average length of time from transplanting until curd visibility in +/+, +/–, and –/– plants was 88.2, 84.6, and 80 days, respectively. The differences in curding time observed in the A×B F_2_ population are relatively small compared with the differences between parents A and B. This may be partly due to the fact that parent lines A and B were not isogenic, meaning that in addition to *BoFLC2,* other genes that affect flowering time would also be segregating and influencing the phenotype of the F_2_ population, resulting in less extreme phenotypes than in the parent lines. In the C×D F_2_ population, the average length of time from transplanting until curd visibility in +/+, +/–, and –/– plants was 94.5, 88, and 82.1 days, respectively. In the C×D F_2_ population, comparison of developmental stage between genotypic classes was conducted at a fixed point of 117 days after transplanting, with Fig. 2B showing significant (*P*<0.0001) differences between the three genotypes. Developmental stage was not able to be measured in the A×B population owing to its susceptibility to attack by saprophytic pathogens following curd initiation.

The importance of *BoFLC2* to the flowering phenotype in both F2 populations is clear. The gene seems to function in a dosage-dependent manner with curd formation delayed in an additive manner by additional copies of the functional allele. Similarly, as the number of functional alleles increased, the developmental stage at a fixed time point was observed to decline. The incomplete dominance exposed in this study contrasts with other studies that suggested that the annual habit is dominant over the biennial habit (Okazaki *et al.*, 2007). Applying the formula σ^2^_phenotype_=σ^2^_genotype_+σ^2^_environment_ (c.f. Supplementary Table S3) to the observed variance under a model of normal distribution would roughly equate to the *BoFLC2* gene explaining 40% of total phenotypic variance in flowering time of the C×D F_2_ population, and 65% of the genetic variance in flowering time, where flowering time is based on the developmental stage of development 117 days after transplanting, and assuming environmental variance in the F_2_ population is the same as the average of the variance in the homozygous parent lines (parental lines were used as environmental controls).

It seems likely that the remaining variation may be explained by other flowering time genes. For example, Irwin *et al.* (2012) suggested that allelic variation at the locus of the brassica orthologue *FRIGIDA* (*BoFRIa*) may be responsible for variation in *B. oleracea* flowering time. A recent QTL study by Uptmoor *et al.* (2012) using a *B. oleracea* population derived from a cross between late/early flowering types indicated that flowering time variability was primarily due to differences in vernalization response. However, neither parent used to generate that population carried functional alleles of *BoFLC2* and no other *BoFLC* genes were found to co-segregate with flowering time. The authors raise the possibility that *FLC-*independent pathways are responsible for the vernalization response seen in their population, and this may also be the case in the populations generated in the study presented here. An attempt was made to map other *B. oleracea* flowering time orthologues including*, BoFLC1, BoFLC3, BoFLC5, BoFT, BoVIN3, SOC1, FRI, BoREM1, CCE1, APETALA1* (*AP1*)*, CAULIFLOWER* (*CAL*), and *LEAFY* (*LFY* or *BoFH*), but no polymorphisms suitable for marker development were identified.

Curd size was recorded throughout the observation period in C×D F_2_ plants. In the first week of measurement (78 days after planting), the average curd size of –/– plants was 11.1cm, greater than that of the +/– plants (5.2cm), which in turn was greater than the average curd size of +/+ plants (2.0cm; see Fig. 2C). In the –/– genotype, curd size increased steadily before reaching a plateau. The other two genotypes seem as though they would also follow this general trend, but recording ceased before a clear plateau was reached. Despite significant differences (*P*<0.0001) in average maximum observed curd size (–/–=26.7cm; +/–=25.0cm; +/+=22.1cm) it seems that all three genotypes were tracking to reach a roughly similar maximum size, and by fitting quadratic trend lines to the data, similar maximum average curd diameters are predicted for each genotype (24.9cm, 23.1cm, and 25.9cm for –/–, +/–, +/+, respectively). This, along with significant variability in curd size at the point of bolting within each genotype, suggests that *BoFLC2* has little bearing on final curd size. However, whereas the homozygous mutant *boflc2* lines would be predicted to reach this maximum size after 108 days, the heterozygotes would require a further 6 days to reach this predicted size, and the homozygous functional lines would require yet another 26 days. Differences in curd initiation date were taken into account through the use of a separate slopes model. A comparison of the slope between average Y values at the first two time points in the –/– line and the average Y values at the first three points in the +/– and +/+ lines (Fig. 2C) was made. This revealed that the slopes (indicating rate of curd growth) of the three genotypes were significantly different (*P*=0.0427). The rate of curd growth was slower in lines with more functional copies of *BoFLC2.*

### Vernalization regulates *BoFLC2* expression in parallel with curd induction

**Fig. 3.**
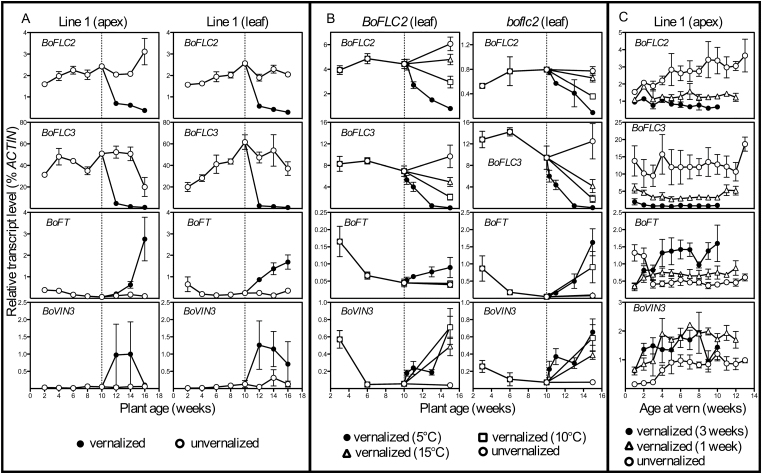
Average *BoFLC2*, *BoFLC3*, *BoFT*, and *BoVIN3* expression. The timing of vernalization treatment is indicated by a dashed line. Bar (A) shows average gene expression in apex and leaf tissue of late-flowering parent line 1 (c.f. Supplementary Table S1). For each point, *n*=3. (B) Average gene expression in leaves of *BoFLC2* and *boflc2* parent lines. Each series represents the average expression of four different parent lines (one replicate per line) within the *BoFLC2* or *boflc2* genotype. Panel (C) shows average gene expression in apical tissue of cauliflower line 1 plants vernalized at different ages for different durations. Data is plotted against plant age at the time of vernalization treatment, or simply against plant age in the case of unvernalized controls. For each point, *n*=3 except for *BoFLC2* week 10 and *BoVIN3* weeks 4 and 9 where *n*=2. In all graphs, error bars represent the standard error of the mean.

In view of the important role of *BoFLC2* in vernalization-mediated flowering, we next examined how *BoFLC2* is regulated by chilling and how this might be related to curd induction behaviour. Three sets of controlled-environment experiments were conducted to examine physiological and molecular responses to vernalization. In the first, a late-flowering *BoFLC2* parent line (line 1) was subjected to vernalization when plants were ten weeks old with gene expression measured two, four, and six weeks after vernalization (Fig. 3A). In the second, ten-week-old *BoFLC2* and *boflc2* parent lines were exposed to vernalization at a range of temperatures (5 °C, 10 °C, and 15°C), and expression was measured 2, 7, 21, and 35 days after vernalization (Fig. 3B). In the third, parent line 1 plants aged 1–12 weeks were exposed to 1- and 3-week 5 °C vernalization treatments (Fig. 3C).

In the first experiment, a direct relationship between vernalization duration and curd initiation/development was observed, with longer periods of vernalization associated with more rapid curding and curd development upon return to glasshouse conditions. In the second experiment using *BoFLC2* and *boflc2* parents, numerous instances of reversion from floral to vegetative growth were recorded when vernalized plants were subsequently returned to 22 °C cabinets. Given the capacity of these post-vernalization conditions to induce such floral reversion, it is also possible that devernalization may have significantly delayed curd induction in some cases. Despite this complication, clear differences in reproductive behaviour were observed between *BoFLC2* and *boflc2* genotypes (Supplementary Fig. S3). Plants carrying the *boflc2* allele were capable of producing curds without any chilling; and yet, a vernalization response was evident, with all vernalization durations increasing the percentage of plants with reproductive structures. Curd induction in *BoFLC2* lines was unaffected by 5 °C vernalization of 2, 7, and 21 days, but vernalization for 35 days promoted curd initiation, with decreasing temperatures amplifying this effect.

Many of the phenotypic differences between vernalization treatments and between flowering types were strongly linked to *BoFLC2* expression behaviour. It was shown for the first time that the basic down-regulation of *BoFLC2* is conserved in cauliflower, with transcript levels markedly reduced by as little as one week of vernalization (Fig. 3B). Colder temperatures and longer periods of vernalization amplified this effect. This transcriptional vernalization response occurred independently of plant age (Fig. 3C). In this regard, *BoFLC2* vernalization responsiveness is more closely aligned to that of seed-vernalization-responsive members of the Brassicaceae, such as *A. thaliana*, *B. rapa*, and *B. napus,* than the even more closely related cabbage, whose *BoFLC* expression is unaffected by vernalization at young ages (Lin *et al.*, 2005). Although expression responses of these two genes were very similar in both *BoFLC2* and *boflc2* varieties, the overall level of *BoFLC2* transcript was approximately five-times higher than that of the premature termination codon (PTC)-carrying *boflc2*, probably owing to nonsense-mediated decay (NMD) of the *boflc2* mRNA (Fig. 3B).

### The transcriptional regulation of three additional *Arabidopsis* vernalization pathway homologues is conserved in *B. oleracea*

Having shown that the molecular regulation of *BoFLC2* by vernalization is consistent with its role as an important regulator of flowering time, we next isolated and characterized the transcriptional behaviour of *BoVIN3*, *BoFT*, and *BoFLC3* to gain a greater understanding of the brassica vernalization pathway.

A homologue of the upstream epigenetic repressor of *FLC*, *VIN3*, was isolated for the first time in a brassica crop species (Supplementary Fig. S4). Our results support the idea that the function of this gene is conserved in cauliflower, with *BoVIN3* consistently up-regulated in response to vernalization in all plant tissues, and with no significant differences observed between early- and late-flowering parent lines (Fig. 3). This suggests that the expression response of this *BoVIN3* allele to vernalization is not a major contributing factor to variability in vernalization responsiveness or flowering time in the cauliflower lines investigated. In contrast to the *Arabidopsis* model, in which prolonged cold temperatures (approx. 20 days at 2–4 °C) are required to induce expression (or more accurately, relieve the repression; Kim *et al.*, 2010) of *VIN3*, results from this study show that *BoVIN3* is significantly up-regulated by as little as 2 days of 5 °C vernalization and that there is little difference between expression levels in plants vernalized for 2, 7, and 21 days at 5 °C (Fig. 3B). *BoVIN3* expression in leaves increased substantially after 35 days of vernalization. It is possible that the up-regulation of *BoVIN3* observed following shorter vernalization treatments (i.e. those less than 35 days) is insufficient to elicit stable down-regulation of *BoFLC* (i.e. down-regulation that is maintained even in the absence of the cold stimulus), accounting for the lack of curd initiation in *BoFLC2* plants. Lin *et al.* (2005) raised the possibility that *BoVIN3* may be involved in the differential response of seed as opposed to plant vernalization in cabbage, suggesting that *BoVIN3* may only be up-regulated during vernalization of mature cabbages, but not in vernalized seedlings. However, Fig. 3C shows that in this study, *BoVIN3* transcription was up-regulated by vernalization regardless of plant age.

*BoFT*, the putative downstream target of *BoFLC2* was characterized in cauliflower for the first time, and found to be up-regulated in response to vernalization (Fig. 3). Cauliflower *BoFT* was up-regulated in both leaves and apices (Fig. 3A), unlike cabbage *BoFT*, which was unaffected by vernalization in leaf tissue (Lin *et al.*, 2005). The overall level of *BoFT* expression was approximately tenfold lower in *BoFLC2* parent lines, reflecting strong suppression by *BoFLC2* (Fig. 3B). In late-flowering parent lines, the up-regulation of *BoFT* in response to vernalization increased with plant age, with younger plants showing little vernalization response (Fig. 3C). This suggests that some factor other than *BoFLC* is contributing to cauliflower juvenility given that *BoFLC2* is significantly (*P*=0.05) down-regulated by three weeks of vernalization independently of plant age (except for *P*=0.083 at week 10 where one replicate was excluded owing to qPCR failure). It is possible that delayed *BoFT* responsiveness is mediated by a transcriptional activator, as opposed to putative negative regulators such as *BoFLC* and *BoVIN3*.

*BoFLC3* expression patterns were similar to those of *BoFLC2.* Whereas Lin *et al.* (2005) found that *BoFLC3* transcript levels were too low to detect using a probe derived from the 3’-UTR of *BoFLC3*, this study showed that *BoFLC3* is strongly expressed in cauliflower, at a level substantially higher than *BoFLC2*, and is also regulated by vernalization in a similar manner to *BoFLC2* (Fig. 3). Therefore, it seems probable that *BoFLC3* could make at least a minor contribution to the differences in timing of curd formation in *boflc2* parent lines following vernalization. Furthermore, recent studies have shown that in species with expanded flowering gene families, transcriptional cross-regulation between different members of the same family may occur (e.g. Hecht *et al.*, 2011); these results suggest that there is no such cross-regulation between *BoFLC3* and *BoFLC2,* with *BoFLC3* regulation entirely independent of *BoFLC2* expression and functionality.

### Correlations between gene expression and reproductive state are preserved under field conditions

Although the molecular basis of vernalization-dependent flowering time regulation in *Arabidopsis* has been intensively studied under controlled conditions, comparatively little is known about flowering gene expression control in more complex, natural environments. Indeed, it has recently been shown that gene regulation in a fluctuating environment is substantially different to that observed in the homogenous and controlled environment of the laboratory (Richards *et al.*, 2009). In 2010, results from a two-year census of the seasonal expression of *FLC* in an perennial population of outdoor-grown *Arabidopsis halleri* were reported, showing that seasonal trends of temperature were detected at the level of *FLC* regulation (Aikawa *et al.*, 2010). However, apart from this study, there are very few reports of the measurement of seasonal/temporal changes in gene expression under natural conditions.

Having examined gene expression in response to artificial vernalization treatments, we next investigated the transcriptional dynamics of flowering genes in pot-grown cauliflower plants exposed to natural, fluctuating temperature conditions University of Tasmania’s Horticultural Research Centre (Sandy Bay, Tasmania). To avoid the problems associated with poor genotype–environment matches encountered in earlier outdoor trials, sowings of late-flowering parent line 51 and early-flowering line 48 were staggered to synchronise the transition from vegetative to reproductive growth in the two lines.

**Fig. 4.**
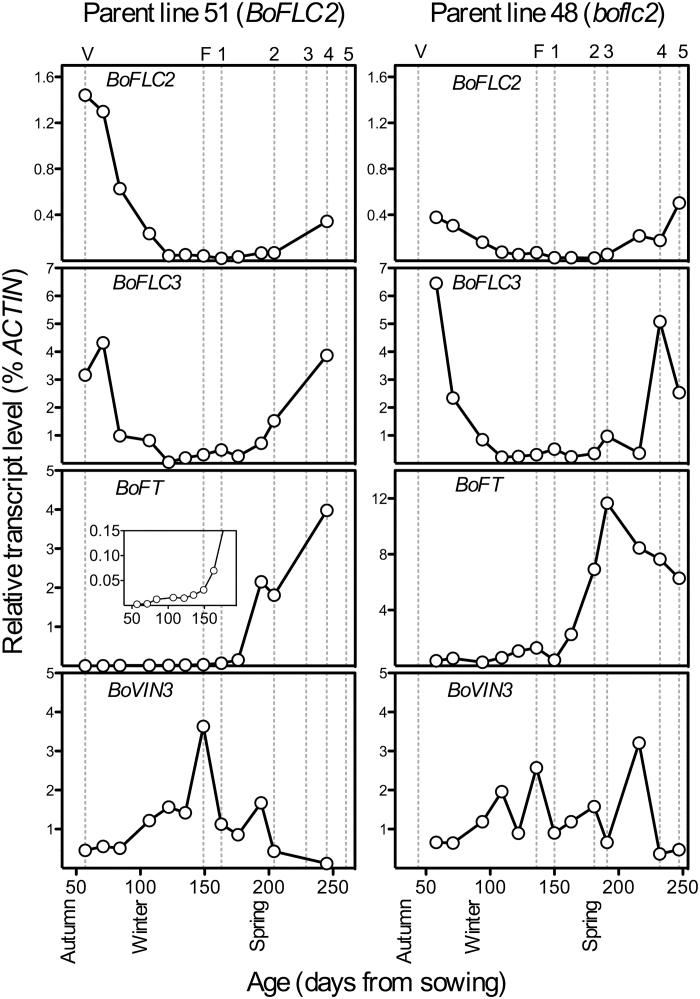
Gene expression throughout autumn, winter, and spring in apical tissue of cauliflower parent lines 51 *(BoFLC2*) and 48 (*boflc2*). Inset: detail of *BoFT* expression during early growth. Values are based on one replicate only. The developmental stage of both parent lines is shown above each set of graphs, where V=vegetative; F=fat, expanded apex without any discernible curd structure; and 1–5=developmental stages according to Supplementary Fig. S1. Parent line details are provided in Supplementary Table S1.

Levels of *BoFLC2* mRNA in apices of the late-flowering line 51 were initially high during early vegetative growth in mid-autumn (Fig. 4). Expression was rapidly down-regulated throughout late autumn and early winter, reaching minimum values approximately 120 days after sowing, nearly a month before a swollen apical bud was detected. Expression remained low throughout early curd development, eventually rising slightly in curds with distinct floral buds. Fluctuations in temperature throughout the seasons had little effect on expression, suggesting the involvement of a ‘buffering’ mechanism in *FLC* regulation that allows seasonal cues to be extracted from the background noise of fluctuating temperatures. Consistent with earlier findings, expression of *BoFLC2* in lines carrying the *boflc2* mutation was greatly diminished in the non-functional line 48, but the same basic profile was recorded, albeit with more pronounced up-regulation as curds elongated and flowered during the spring months. *BoFLC3* patterns were similar.

Figure 4 shows that initial *BoFT* expression levels in line 51 were very low throughout autumn and early winter. A consistent, but slight up-regulation representing a 13-fold increase in mRNA levels from 57 days after sowing to 149 days after sowing may be seen when the data is viewed at a smaller scale, but it is not until 163 days after sowing, when visible curds were first detected, that up-regulation of *BoFT* accelerates substantially. However, these changes in *BoFT* expression are minor compared with the marked up-regulation that occurs during curd expansion, and the subsequent up-regulation associated with the development of floral buds throughout spring. The main *BoFT* up-regulation occurred nearly a month after the *BoFT*-inhibitor *BoFLC2* reached its lowest point. In the younger, early-flowering parent line 48, a similar profile was generated, with a marked increase in *BoFT* expression also observed immediately following curd formation, before mRNA levels taper off during later curd development.

*BoVIN3* expression in line 51 generally increased throughout autumn/winter before a peak 149 days after sowing, when ‘fat’ apices were first noted (Fig. 4). After this point, *BoVIN3* expression trended downwards. In the *boflc2* line, *BoVIN3* expression was highly erratic from week to week, and there does not seem to be any connection between the stage of reproductive development and *BoVIN3* expression.

This long-term gene expression study provides the first realistic profile of gene expression under natural conditions in cauliflower, and is an essential link between controlled-environment experiments with artificial vernalization treatments and ‘real-world’ application. Importantly, it also indicates robust regulation of the long-term control of *BoFLC* and *BoFT* in both lines, with episodic warm temperatures throughout the autumn and winter months seeming to have little influence on the overall direction of gene regulation. This suggests that the epigenetic memory of winter in these two lines is sufficiently long to buffer against short-term temperature fluctuations (c.f. Aikawa *et al.*, 2010).

**Fig. 5.**
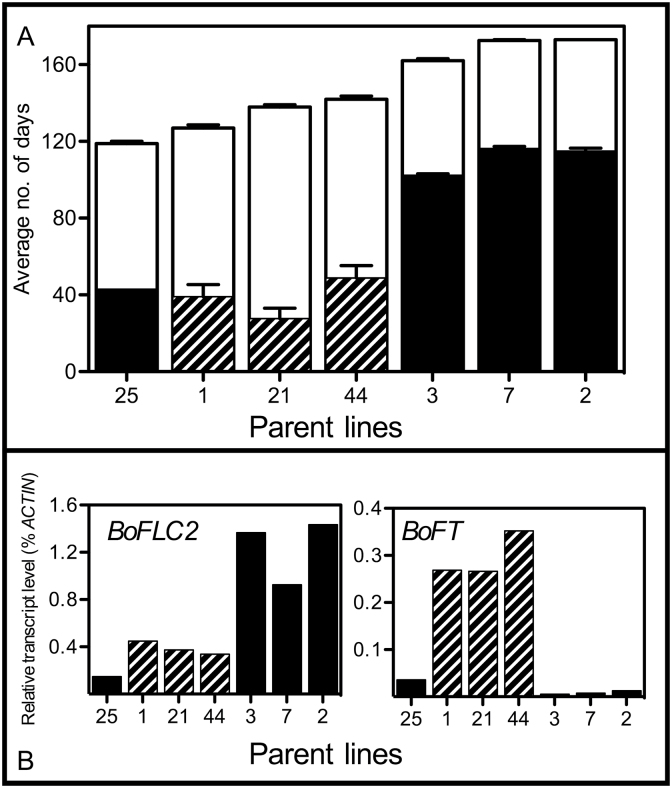
Reproductive behaviour of *BFLC2* cauliflower parent lines grown under field conditions, and relative expression of apical *BoFLC2* and *BoFT* at a fixed time point. In (A), black bars represent the average number of days to visible curd formation. Striped bars indicate romanesco varieties. White bars indicate the average number of days to anthesis. Values for the average number of days to both curding and anthesis are counted from the 13 June and are the average of up to 30 plants (three replicates with ten duplicates in each replicate), with dead or missing plants excluded from the analysis. Plants that had not curded or flowered by the end of the observation period were assigned a maximum score of 180 days. Error bars represent the standard error of the mean. (B) *BoFLC2* and *BoFT* gene expression at a fixed point before curd induction (13 June). Striped bars indicate romanesco varieties. Parent line details are provided in Supplementary Table S1.

In the Forthside field trial, two distinct groups were identified, in terms of reproductive behaviour. The “very late” parent lines 2, 3, and 7 formed curds and subsequently went on to flower much later than the other group of *BoFLC2* lines which comprised romanesco lines 1, 21 and 44 and “Late” line 25 (Fig. 5A). As seen in Fig. 5B, there was a strong positive correlation between *BoFLC2* expression at a fixed time point before curd initiation and both the average number of days to curding (r=0.892, *P*=0.003) and the average number of days to flowering (r=0.880, *P*=0.004). These results provide strong evidence in support of the idea that *BoFLC2* expression in vegetative plants is a reliable indicator of curding and flowering time in *BoFLC2* parent lines. *BoFT* expression levels in apices at the sampling date were also strongly correlated with curding time (r=–0.760, *P*=0.024), but not with flowering time.

Although interest in the development of genetically informed models of progression towards flowering has increased in recent years (Uptmoor *et al.*, 2008; Wilczek *et al.*, 2009), the concept of using gene expression data to monitor a plant’s developmental status has not been explored to any significant extent. These results indicate that the correlations described between gene expression and reproductive state under controlled conditions are also valid under field conditions and illustrate the potential for such expression assays to form the basis of molecular tools for understanding and predicting flowering in dynamic and complex production environments.

## Conclusion

Using an integrated physiological, genetic, and molecular approach, we have demonstrated the role of *BoFLC2* as a key determinant of flowering time within the annual *B. oleracea* var. *botrytis* group. An association approach provided evidence that variation at the *BoFLC2* locus is an important determinant of reproductive behaviour, and this hypothesis was strengthened with a more specific study involving segregating populations. Regulation of *BoFLC2* expression in response to vernalization is consistent with the FLC function observed in the canonical *Arabidopsis* vernalization pathway, and transcriptional profiles of both upstream and downstream genes serve to support this view; however, a considerable amount of work remains to fully elucidate the cauliflower flowering pathway. In particular, the ancestral polyploid nature of this species means that there are likely to be multiple additional undiscovered genes even within the families of those genes investigated here that also interact with one another and make significant contributions to the floral transition. The validation of gene expression behaviour under outdoor conditions raises the potential for measurements of relative transcript level to form the basis of assays that could be used to predict curd induction or flowering in production environments.

## Supplementary data

Supplementary data are available at *JXB* online.

Figure S1. Curd development key.

Figure S2. Distribution of *BoFLC2* genotypes across the five cauliflower flowering time classes.

Figure S3: Percentage of curds initiated following vernalization of *BoFLC2* and *boflc2* parent lines.

Figure S4: Alignment of *BoVIN3* amino acid sequence with *B. rapa* and *A. thaliana* homologues.

Table S1. Brassica variety/flowering classifications.

Table S2. Primer combinations used for gene isolation and RT-qPCR.

Table S3. Calculation of the contribution of *BoFLC2* to phenotypic and genetic variance.

Supplementary Data
