# Supplementary material for: The role of BoFLC2 in cauliflower (Brassica oleracea var. botrytis L.) reproductive development
Source: J Exp Bot. 2014 Oct 28;66(1):125–35. doi: 10.1093/jxb/eru408 (PMC4265156; doi:10.1093/jxb/eru408)
Supplement: Supplementary Data [file supp_66_1_125__index.html]

The role of BoFLC2 in cauliflower (Brassica oleracea var. botrytis L.) reproductive development — The role of BoFLC2 in cauliflower (Brassica oleracea var. botrytis L.) reproductive development — Supplementary Data 

# The role of *BoFLC2* in cauliflower (*Brassica oleracea* var. *botrytis* L.) reproductive development

## Supplementary Data

Data files

**Files in this Data Supplement:**

- Supplementary Data - Supplementary Data
