# Supplementary material for: The role of BoFLC2 in cauliflower (Brassica oleracea var. botrytis L.) reproductive development
Source: J Exp Bot. 2014 Oct 28;66(1):125–35. doi: 10.1093/jxb/eru408 (PMC4265156; doi:10.1093/jxb/eru408)
Supplement: Supplementary Data [file supp_eru408_jexbot128447_file001.pdf]

SUPPLEMENTARY DATA

The Role of *BoFLC2* in Cauliflower (*Brassica oleracea* var. *botrytis* L.) Reproductive Development

Stephen Ridge, Philip H. Brown, Valérie Hecht, Ronald G. Driessen and James L. Weller

List of supplementary figures and tables:

Table S1: Brassica variety/flowering classifications

Figure S1: Curd Development Key

Table S2: Primer combinations used for gene isolation and RT-qPCR

Figure S2: Distribution of *BoFLC2* genotypes across the five cauliflower flowering time classes

Table S3: Calculation of the contribution of *BoFLC2* to phenotypic and genetic variance

Figure S3: Percentage of curds initiated following vernalization of *BoFLC2* and *boflc2* parent lines

Figure S4: Alignment of *BoVIN3* amino acid sequence with *B. rapa* and *A. thaliana* homologues

CAULIFLOWER PARENT LINE	CAULIFLOWER FLOWERING CLASS	CAULIFLOWER PARENT LINE	CAULIFLOWER FLOWERING CLASS
1	Late	28	Very Late
2	Very Late	29	Med Late
3	Very Late	30	Early
4	Very Late	31	Early
5	Very Late	32	Early
6	Med Early	33	Med Late
7	Very Late	34	Med Early
8	Med Early	35	Early
9	Late	36	Med Early
10	Early	37	Early
11	Very Late	38	Early
12	Very Late	39	Early
13	Late	40	Early
14	Late	41	Med Late
15	Very Late	42	Med Early
16	Late	43	Med Early
17	Late	44	Med Late
18	Med Late	45	Med Early
19	Late	46	Late
20	Med Early	47	Med Early
21	Med Late	48	Med Early
22	Very Late	49	Med Late
23	Med Early	50	Med Late
24	Late	51	Late
25	Late	52	Med Late
26	Very Late	53	Med Late
27	Med Late	54	Early

Table S1. Brassica variety/flowering classifications. The 54 cauliflower parent lines were grouped into five flowering classes by Rijk Zwaan breeders, based on time from planting to full flower under field conditions in Fijnaart, Noord Brabant, Netherlands: Class 1: Early (50-70 days); Class 2: Medium Early (70-80 days); Class 3: Medium Late (80-120 days); Class 4: Late (120-180 days); Class 5: Very Late (180-250 days).

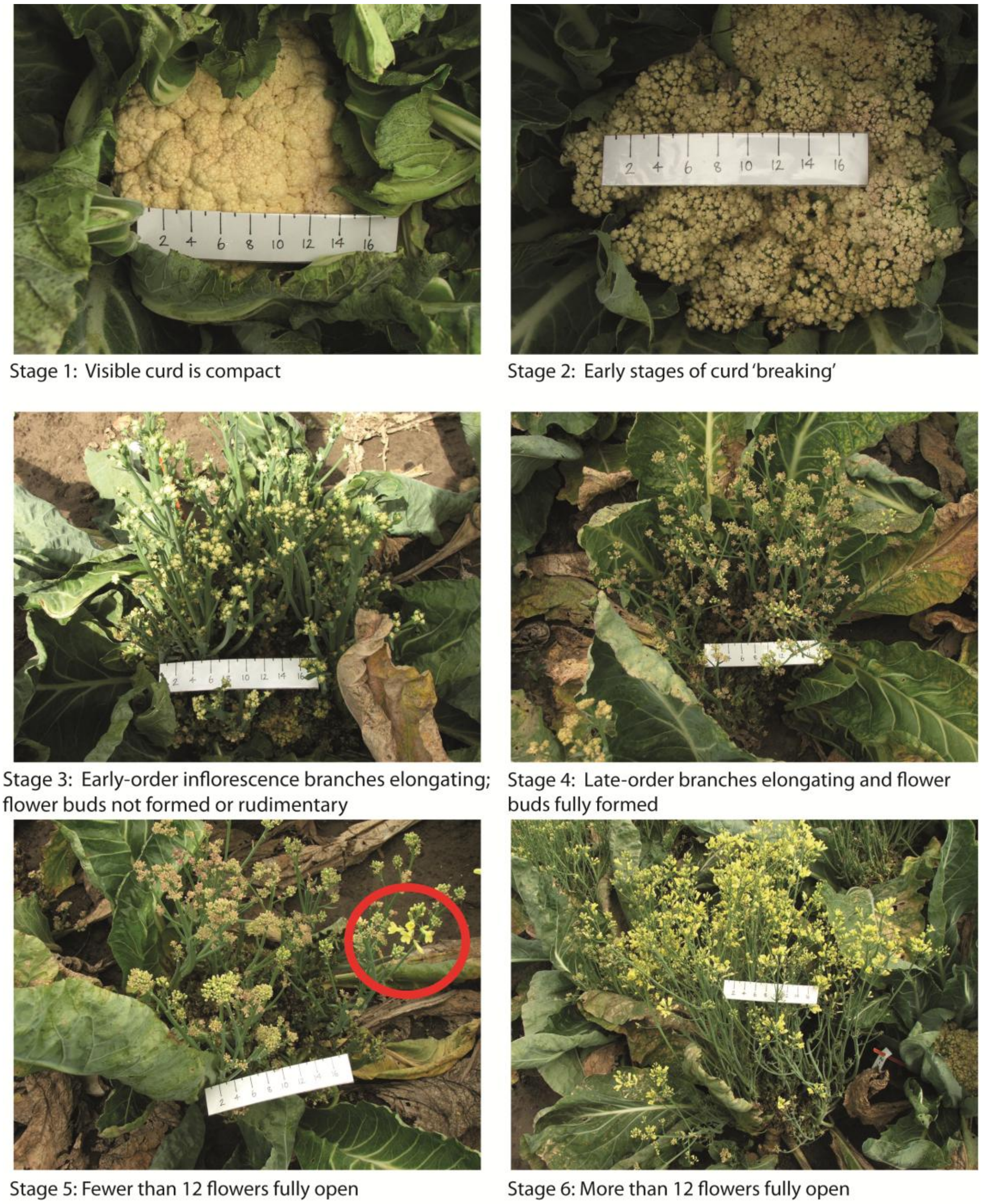

Fig. S1. Curd Development Key. Stage 1 corresponds to a curd that is visible without damaging excavation amongst apical leaves, and that is compact, with no discernible elongation of peduncles. Stage 2 corresponds to a 'loose' curd that is beginning to 'break'. Stage 3 corresponds to a curd whose early-order inflorescence branches are elongating, but where individual flower buds are not yet formed, or are rudimentary. Stage 4 curds have fully-formed flower buds (similar to the individual buds of a broccoli head). Stage 5 corresponds to a curd that has one or more flowers fully open (circled), but less than twelve flowers fully open. Stage 6 is the highest developmental category, and curds have more than 12 flowers open, and may have started to produce fruit.

GENE	FORWARD PRIMER (5'-3')	REVERSE PRIMER (5'-3')	Tm	PRODUCT SIZE (bp)	PURPOSE
BoFLC1	GGCTTTTGATTATGGACAAACC	AACCCAACTTGGAATCAAACC	59	603	Checking for SNPs in 5'UTR
BoFLC1	GGAGTCCATCTTTCCACGTT	TGATCTTGAGGTCCGGTTTC	59	717	Checking for SNPs in 5'UTR
BoFLC2	AGGGCCTAGAGGGCATACAT	TTTTGAGGCTCTCGACACAA	59	571-572 (gDNA)	Partial isolation and CAPS (PvuII)
BoFLC2	CGAAGTATGGTTCACACCATGAGC	CGGAGAGGGCAGTCTCAAGGTGGTT	58	122-123	qPCR
BoFLC3-2	AGCAGAAACGGGAACCTACA	CATTGCCATTTACTGCATCG	59	812	Checking for SNPs in 5'UTR
BoFLC3-2	CCTCCTCCGGAAAGCTCTACAGC	TGTCCACGCTTACACCAACGAC	58	202	qPCR
BoFLC4-1	GAAATATGGAAAGCGGGTGA	AGAGGTGATGCGCCTAGAAA	59	744-986	Checking for SNPs in 5'UTR
BoFLC4-1	GGGATTGCGCAAAATTCTAA	GCGTGGACGGCTAGTGTATT	59	610-852	Checking for SNPs in 5'UTR
BoFLC5	CCATTCGAGTTCGGATATTG	CGATGCGTTTTAACGACAAG	59	252	Checking for SNPs in 5'UTR
BoFLC5	TGATGAAGGAAGACCCTTGG	TTCTCTATCATGGCGGTTCC	59	634	Checking for SNPs in intron 1
BoFLC5	ATCGGCAATCGTTGTGTGTA	CCCAAGACAAAAGACCCAAG	59	773	Checking for SNPs in intron 1
BoFT	TCAACACAGAGAAACCACCTG	CATCACCGTTCGTTACTCG	56	675	Isolation
BoFT	GCCAAAGAGAGGTGACAAATGG	CCAACCAATGGAGATATTCTCGT	58	171	qPCR
BoVIN3	TAGTGATCTTCTCGGATGCTG	GCCTCATCCATGAGTTCCAA	56	150	qPCR
BoACT	GAGATTCAGGTGTCCAGAGG	GCTGTGATCTCTTTGCTCATACGGTC	60	196	qPCR

Table S2. Primer combinations used for gene isolation and RT-qPCR. Product size is based on cDNA unless otherwise indicated.

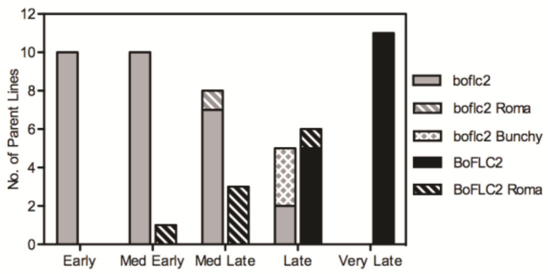

Fig. S2. Distribution of *BoFLC2* genotypes across the five cauliflower flowering time classes. *BoFLC2* lines are denoted by black bars and *boflc2* lines are denoted by grey bars. The striped component of each category indicates the proportion of lines that are Romanesco (denoted 'Roma' in the legend) and the spotted component represent the proportion of lines that formed 'heads' with tightly-packed leaves (denoted 'Bunchy' in the legend). Flowering classes are based on **Table S1**.

One Way ANOVA	Sum of Squares	Degrees of Freedom	Error Mean Square	P Value
**F_2 Residual Variance ($\sigma^2_{genetic\ background}$+$\sigma^2_{environment}$)**	363.73	357	1.02	<0.001
**F_2 Total Phenotypic Variance ($\sigma^2_{genotype}$+$\sigma^2_{environment}$)**	606.26	359	1.69	<0.001
**Parent Residual Variance ($\sigma^2_{environment}$)**	34.19	52	0.66	<0.001

Table S3. Calculation of the contribution of *BoFLC2* to phenotypic and genetic variance. The contribution of *BoFLC2* to phenotypic and genetic variance in the CxD F_2 population was calculated using the formula $\sigma^2_{phenotype} = \sigma^2_{genotype} + \sigma^2_{environment}$ under a model of normal distribution. The $\sigma^2_{genotype}$ component was further divided into σ^2_{BoFLC2} and $\sigma^2_{genetic\ background}$. To find σ^2_{BoFLC2}, F_2 residual variance (1.02) was subtracted from F_2 total phenotypic variance (1.69) = 0.67. To find $\sigma^2_{genotype}$, parental residual variance (0.66) was subtracted from total variance (1.69) = 1.03. To calculate the contribution of *BoFLC2* to total phenotypic variance in flowering time, σ^2_{BoFLC2} (0.67) was divided by F_2 total phenotypic variance (1.69) = 40%. To calculate the contribution of *BoFLC2* to genetic variance in flowering time, σ^2_{BoFLC2} (0.67) was divided by $\sigma^2_{genotype}$ (1.03) = 65%. Individual plants were excluded where flowering time had been delayed due to disease or where plants had died.

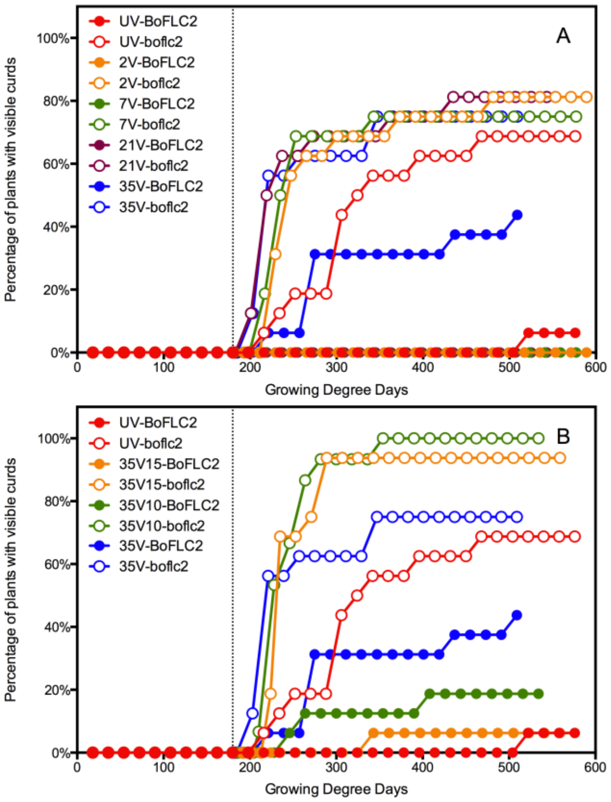

Fig. S3. Percentage of curds initiated following vernalization of *BoFLC2* and *boflc2* parent lines. (A) and (B) show the effect of vernalization length and temperature, respectively, on visible curd induction. The initial digits in the treatment code indicate the duration (days) of vernalization (e.g. 2, 7, 21, 35, or 'UV' if plants were unvernalized). Vernalization temperature was 5°C in all treatments except two; for these 10°C and 15°C treatments, the vernalization temperature is also included in the treatment name. Eight different flowering types were evaluated; four *BoFLC2* lines, and four *boflc2* lines. Percentage values shown here represent the % of plants with curds in each of these combined two groups (data for individual lines not shown). Each of the four lines in the *BoFLC2* and *boflc2* groups had four replicates, meaning that these percentage values relate to the % of a group of 16 samples. Data is plotted against Growing degree-days (GDD) to account for differences in plant development arising from different vernalization treatments. GDD were calculated using a heuristic, standard formula (e.g. McMaster and Wilhelm, 1997), modified to allow for the fact that both UV and V temperatures were constant (i.e. with no day/night or max/min temperature): $T_{DAILY} - T_{BASE}$, where T_{DAILY} = growth cabinet temperature for a 24 hr period and T_{BASE} = base temperature at which cauliflower growth had been observed to cease; in this case, 4°C. The dotted lines represent the point at which plants were vernalized.

```
                     *        20         *        40         *        60         *        80
BrVIN3 : -------------------------------------------------IHALSNQPEEASELLNSWSRDEIMKIICAEM :  31
BoVIN3 : ---------------CGPRRRHGSFFISTCRVHEFPKKKGLSVSERRELIHALSKQPEEASELLNSWSRDEIMKIICAEM :  65
AtVIN3 : MQAASLSKIWRFDGNVGPENMDSSSFEDN-ECIETCKPNVLNVSERRELIHALSNQPEEASELLNSWSRNEIMKIICAEM :  79

                     *       100         *       120         *       140         *       160
BrVIN3 : GKERKYTGLAKPKLIETLLKLVSRPLGETSRPDRKNSKKKRK-TTSYIICCENVACRAALGTEDTFCRKCSCCVCQNYDE : 110
BoVIN3 : GKERKYTGLAKPKLIENLLNLVSRPLGETSCPNRKSSRKKQKTTTSYIICCENLACRAALGSEDTFCRRCSCCVCQNFDE : 145
AtVIN3 : GKERKYTGLNKPKLIENLLNLVSRPLGETSCSDRRNSRKKEKKMIGYIICCENLACRAALGCDDTFCRRCSCCICQKFDD : 159

                     *       180         *       200         *       220         *       240
BrVIN3 : DKDPSLWITCEACGVSCHLECALEQERYGIGCDDDEVGRALDGRFYCAFCGKDNDLLGCWRQQVKVAKETQRVDVLCYRV : 190
BoVIN3 : NKDPSLWIACEGCGLSCHLECALKQDGYGIGYND--------GSFHCVFCGKDSDLLGCWRKQVKVAKETGRVDVLCYRV : 217
AtVIN3 : NKDPSLWLTCDACGSSCHLECGLKQDRYGIGSDD------LDGRFYCAYCGKDNDLLGCWRKQVKVAKETRRVDVLCYRL : 233

                     *       260         *       280         *       300
BrVIN3 : SLGQKLLRGTRKYRYLLELMDEAV------------------------------------------ : 214
BoVIN3 : SLGQKLLRGTRRYQNLLELMDEAVKKLEGDMGPLVSWDMKMAREIVNRLASGSQVQKLCL------ : 277
AtVIN3 : SLGQKLLRGTTKYRNLLELMDEAVKKLEGDVGPLSGWAMKMARGIVNRLSSGVHVQKLCSQAMEAL : 299
```

Fig. S4. Alignment of *BoVIN3* amino acid sequence with *B. rapa* and *A. thaliana* homologues. *BrVIN3* denotes the *B. rapa* sequence ACR25202 (NCBI), *BoVIN3* is the sequence isolated in this study, and *AtVIN3* is partial Arabidopsis sequence NP_200548 (NCBI).

REFERENCE

McMaster GS and Wilhelm WW. 1997. Growing Degree-Days: One Equation, Two Interpretations. *Agricultural and Forest Meteorology* **87,** 291-300.
